# Capacitive Biosensing of Skin Irritants Using a Lanolin-Based Artificial Stratum Corneum Model

**DOI:** 10.3390/bios15090564

**Published:** 2025-08-28

**Authors:** Chung-Ting Cheng, Yi Kung, Hung-Yu Chen, Kuang-Hua Chang, Richie L. C. Chen, Tzong-Jih Cheng

**Affiliations:** 1 Department of Biomechatronics Engineering, College of Bio-Resources and Agriculture, National Taiwan University, Taipei 106319, Taiwan; b11b02013@ntu.edu.tw (C.-T.C.); d13631001@ntu.edu.tw (H.-Y.C.); kuanghua@ntu.edu.tw (K.-H.C.); rlcchen@ntu.edu.tw (R.L.C.C.); 2 Department of Biochemical Science & Technology, College of Life Science, National Taiwan University, Taipei 106319, Taiwan; 3 Department of Biomechatronic Engineering, College of Science and Engineering, National Chiayi University, Chiayi 60004, Taiwan; yikung@mail.ncyu.edu.tw; 4 Experimental Farm, College of Bio-Resources and Agriculture, National Taiwan University, Taipei 106319, Taiwan

**Keywords:** capacitive sensor, skin irritation, lanolin, artificial stratum corneum

## Abstract

Skin irritation testing is transitioning toward non-animal alternatives that can replicate the functional properties of the human stratum corneum (SC). To address this need, we report a capacitive biosensing platform that integrates a lanolin-based artificial SC (aSC) for rapid, indicator-free detection of chemical irritants. The approach leverages a membrane-bound lipid matrix to detect changes in interfacial capacitance caused by chemical exposure. Among candidate materials, lanolin emerged as the most effective SC mimic, showing reproducible baseline stability and responsive dielectric shifts. The system quantifies barrier integrity through the capacitance change rate (ΔC/Δt), which serves as a real-time indicator of irritation potential. By positioning the biosensor as an analog of the SC and monitoring the dielectric environment during short exposures (7.5 min), we shift the paradigm from endpoint-based biochemical assays to rapid, physicochemical screening. This concept supports the advancement of ethical, scalable testing platforms that reduce reliance on animal or cellular models while maintaining sensitivity to barrier-compromising agents.

## 1. Introduction

The stratum corneum (SC), the outermost layer of the epidermis, functions as a critical barrier regulating water loss and shielding the body from external insults. It comprises corneocytes embedded in a lipid-rich extracellular matrix, primarily composed of ceramides, cholesterol, and free fatty acids [1,2,3,4]. This highly organized structure provides both mechanical strength and hydrophobicity, restricting the permeation of water and xenobiotics [5]. In addition to physical protection, the SC defends against ultraviolet radiation, environmental pollutants, and microbial invasion. Compromised SC integrity—due to chemical, mechanical, or pathological stressors—can lead to increased skin permeability and vulnerability to irritants, often resulting in inflammation or sensitization [6,7].

Skin irritation is a reversible inflammatory response caused by exposure to substances that disrupt barrier integrity. Conventional assessment methods, such as the Draize test [8], involve topical application of chemicals to rabbit skin followed by visual scoring of erythema and edema. While historically important, this approach faces increasing criticism due to its ethical implications and scientific limitations. Rabbit skin differs significantly from human skin in thickness, lipid composition, and permeability, often leading to exaggerated irritation outcomes [9,10]. Consequently, human-relevant, non-animal alternatives are being developed to align with global efforts in animal welfare and regulatory reform.

Among these alternatives, Reconstructed Human Epidermis (RHE) models have gained traction. Comprising cultured human keratinocytes forming a stratified structure, RHE mimics key aspects of epidermal physiology and is validated under OECD Test Guideline 439 for in vitro skin irritation testing [11,12,13]. These models quantify irritation based on cell viability following chemical exposure [14,15,16,17]. While RHE systems provide good sensitivity (90–100%), their specificity (70–85%) is lower [18], often due to the absence of a complete hydrolipid barrier [17]. Lipid deficiencies—particularly the lack of ceramide-rich lamellar structures—limit their ability to fully replicate the barrier properties of native SC, which is crucial in irritation assessment.

Alternative membrane-based methods such as Corrositex^®^, which utilizes a collagen matrix, have been developed for corrosion testing [19], but they are not suited for reversible irritation studies. Other platforms like Irrtection^®^ use keratin-based reagents, though publicly available validation data remain limited [20]. Notably, none of the current in vitro models explicitly incorporate lipid-based barriers analogous to the SC, leaving a gap in replicating key biophysical properties relevant to irritant permeability.

Lanolin, a naturally derived wax from sheep’s wool [21], presents a promising candidate for artificial SC (aSC) models. Composed of esters, fatty alcohols, and fatty acids, lanolin closely mimics the lipid composition of human SC [22,23,24]. It is well known for its emollient and occlusive properties and is widely used in dermatological formulations to enhance skin hydration and barrier repair [25,26]. Its biocompatibility and ability to reduce transepidermal water loss render it suitable for constructing synthetic barrier layers. Previous studies have applied lanolin to mimic SC in drug permeation assays [21], but its application in in vitro irritation testing remains underexplored.

Traditional analytical techniques for assessing barrier integrity in reconstructed epidermal models include transepithelial electrical resistance (TEER) [27], dye penetration assays, immunohistochemistry, and vibrational spectroscopy [28]. While effective, these methods often rely on endpoint measurements, require complex instrumentation (e.g., HPLC, spectroscopy), and are not conducive to high-throughput analysis. There is growing interest in sensor-based systems capable of monitoring barrier function in real time, particularly for early-stage screening of irritant permeation and drug delivery formulations.

Electrical impedance spectroscopy (EIS) has been widely employed for characterizing skin hydration and barrier function [29,30,31,32]. More recently, it has been applied to in vitro models; however, EIS’s reliance on broadband frequency scanning and equivalent circuit modeling limits its temporal resolution and complicates interpretation [33,34]. Other instruments, such as the Corneometer^®^ and SkinChip^®^, measure skin capacitance to assess moisturization but are limited in scope and sensitivity. Although some studies have derived capacitance values from EIS to monitor the effects of surfactants on barrier disruption [35], poor sensitivity has limited its effectiveness in detecting early structural damage to the SC. To date, no known studies have applied capacitive sensors to lipid-based aSC platforms for irritation assessment.

Capacitive sensors, which monitor changes in dielectric properties at the interface, offer several advantages for this application. They are non-invasive, highly sensitive, and capable of real-time monitoring, making them well-suited for detecting subtle disruptions in barrier integrity caused by irritants [34,36]. Capacitance-based detection does not require reagents, dyes, or post-processing steps, simplifying operation and enabling rapid screening.

In this study, we present a novel in vitro skin irritation platform that integrates a lanolin-based aSC with a custom capacitive sensor system. The aSC was fabricated by coating a hydrophilic mixed cellulose ester (MCE) membrane with a uniform lanolin layer, forming a biphasic structure that simulates the SC’s dual hydrophilic–lipophilic nature. This model was mounted on an acrylic sensing chamber with embedded electrodes, allowing continuous measurement of capacitance before and after exposure to test compounds.

The platform was evaluated using a range of known irritants (e.g., SDS, KOH) and non-irritants (e.g., PBS, isopropanol), with distinct capacitance profiles observed for each category. A quantitative metric—the rate of capacitance change (ΔC/Δt)—was introduced to classify irritation potential. Additional experiments were performed to assess the effects of lanolin thickness and aSC configuration on sensitivity and reproducibility. Time-lapse measurements enabled direct observation of dielectric changes during irritant permeation, offering real-time insight into barrier disruption dynamics.

This lanolin-based aSC system provides a low-cost, scalable, and ethically aligned alternative to animal-based assays and complex tissue models. By combining biomimetic barrier properties with sensitive electrical detection, the platform addresses key limitations in current irritation assessment methods. Moreover, its versatility suggests potential for broader applications, including permeability profiling, transdermal drug delivery evaluation, and biointerface interaction studies. With further optimization, this capacitive sensing approach may serve as a valuable tool in toxicological screening and regulatory decision-making.

## 2. Materials and Methods

### 2.1. Apparatus

Capacitance measurements were performed using the Cap-S system (A&A-x6, Alice & Alex, Taipei, Taiwan). Attenuated total reflection-infrared (ATR-IR) spectra were acquired using an IRAffinity-1S spectrometer (Shimadzu, Kyoto, Japan) with a QATR 10 single-reflection diamond crystal ATR accessory. A LCR meter (LCR819, GW-INSTEK, New Taipei City, Taiwan) was used as a reference instrument for capacitance determination.

### 2.2. Materials

Sodium dodecyl sulfate (SDS, 92.0%, #1933-2350) was purchased from Showa Kako (Tokyo, Japan). Potassium hydroxide (KOH, #28616-45) and Glutaraldehyde (GA, 25%, #17025-25) were obtained from Nacalai Tesque (Tokyo and Kyoto, Japan). Polyethylene glycol 400 (PEG 400, BZ8E28) and lanolin anhydrate (99.7%, KB4C83) were purchased from Emperor Chemical Co. Ltd. (Taipei, Taiwan). Isopropanol (#67-63-0) was sourced from Avantor Performance Materials Inc. (Radnor, PA, USA). Sodium dihydrogen phosphate and potassium dihydrogen phosphate were obtained from Nacalai Tesque (Tokyo, Japan). Other analytical-grade reagents were purchased from Sigma-Aldrich (St. Louis, MO, USA). Petrolatum (Vaseline^®^, 100% white petrolatum) was obtained from Unilever (Rogers, AR, USA), and Parafilm^®^ (PM-996) from Dogger (Taipei, Taiwan). Columnar copper rods were sourced from Jianyuan Hardware Co. (Taipei, Taiwan). Polyacetate tape (Scotch^®^ Magic™ Tape) was obtained from 3M (Maplewood, MN, USA). Double-sided tapes (No. 5601, 5603, and 5610) were supplied by Nitto (Tokyo, Japan) and distributed by Jing Chung Material Co., Ltd. (Taipei, Taiwan). Mixed cellulose ester (MCE) filter membranes (A045A047A, ADVANTEC) were purchased from Toyo Roshi Kaisha Ltd. (Tokyo, Japan).

### 2.3. System Design and Configuration

**Figure 1:**
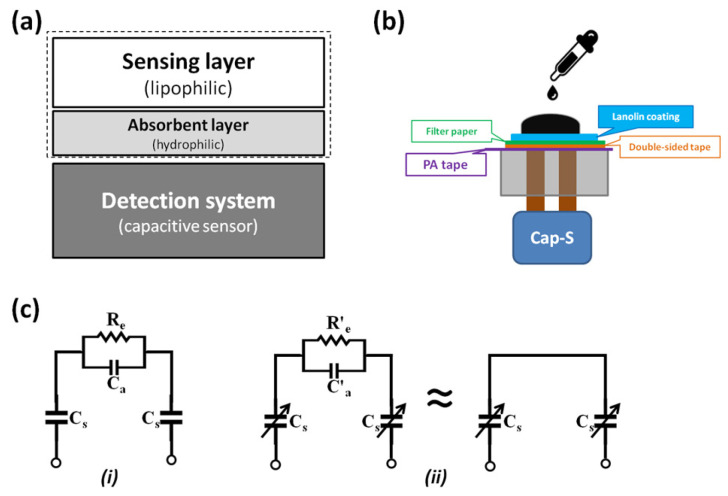
System architecture, configuration, and equivalent electrical circuit model of the capacitive sensing platform for irritation assays. (**a**) System architecture diagram: the lipophilic sensing layer and hydrophilic absorbent layer are stacked to form an artificial stratum corneum (SC) model (dotted square), which is connected to the capacitive measurement system. (**b**) System configuration: a pair of circular electrodes (brown), embedded in an acrylic base, is exposed on the sensing surface. A PA insulating tape (purple) is placed over the electrodes to form the sensing platform, which is then connected to the Cap-S module. An absorbent filter paper (green) is fixed with double-sided tape (orange) and covered with a lanolin sensing layer (blue) to complete the artificial SC. (**c**) Equivalent electrical circuit model: (**i**) Circuit model in air; (**ii**) Circuit model when the sensing surface contacts an electrolyte. Here, C_s_ represents the capacitance of the dielectric layer (including the insulating tape, double-sided tape, and SC layer). C_a_ and R_e_ are the capacitance and resistance of the air path between electrodes, while C′_a_ and R′_e_ represent the capacitance and resistance of the electrolyte path, respectively. This schematic highlights the sensor’s working principle, where dielectric changes at the electrode–coating interface are transduced into measurable capacitance signals, enabling classification of irritant versus non-irritant responses.

A capacitive sensing platform was developed to detect irritant-induced changes in the dielectric properties of an aSC model (Figure 1). The system architecture (Figure 1a) integrates a lipophilic sensing layer and a hydrophilic absorbent layer, replicating the bilayer structure of human SC. These layers are interfaced with a high-resolution capacitive measurement system to enable real-time monitoring of irritant interactions.

The sensing platform comprises a lanolin-coated filter paper affixed with double-sided tape, forming the lipophilic sensing surface. This assembly overlays electrodes insulated by 0.05 mm polyacetal (PA) tape, mounted on an acrylic base. The lanolin layer simulates the SC’s lipid matrix, while the underlying filter paper mimics the moisture-retentive properties of deeper skin layers by absorbing aqueous components of irritants. Upon contact with an irritant, the dielectric characteristics of the lanolin layer change, resulting in a measurable shift in capacitance.

Figure 1b illustrates the complete configuration, including connection to a Cap-S module, offering a nominal resolution of 4 aF and accuracy of 4 fF. Capacitance values are transmitted via USB and recorded using A&A x6-20 software, with a sampling rate of 0.1 s, enabling dynamic tracking of irritant-induced responses. Additional details on hardware and signal acquisition are available in a previous study [37].

### 2.4. Signal Transduction Mechanism and Equivalent Circuit Model

To understand signal behavior, equivalent electrical circuit models were proposed (Figure 1c). In the absence of irritants, the system is modeled as a parallel combination of the aSC capacitor (C_s_), an air capacitor (C_a_), and a resistance path (R_e_) due to ambient conditions. Upon irritant exposure, the model transitions to include modified parameters (C′_e_ and R′_e_), reflecting changes in the sensing layer’s dielectric and conductive properties. These changes were simplified into a two-capacitor series model, facilitating quantification of C_s_ as influenced by irritant interaction.

(1) CTotal=12Cs+1C′a

(2) $${C^{\prime }}_{Total}=\frac{C_{s}}{2}$$

(3) $$C_{s}=\frac{\varepsilon A}{d}$$

where ε is the dielectric constant, A is the electrode area, and *d* is the thickness of the sensing layer. The dielectric stack comprises PA tape, double-sided tape, filter paper, and synthetic films mimicking the SC.

An intact hydrophobic membrane (e.g., lanolin layer) increases the effective dielectric thickness and lowers C_s_, limiting ionic access to the electrode surface. Upon exposure to an irritant such as SDS, membrane disruption allows electrolyte penetration, increasing the local permittivity and ionic mobility at the interface. This reduces the impedance to displacement current, thereby increasing the measured C′_total_.

The change rate, ΔC/Δt, reflects the kinetics of electrolyte ingress and membrane degradation. A faster ΔC/Δt indicates more rapid compromise of the barrier, consistent with irritant action. This can be modeled by a simplified equivalent circuit, where C_s_ and a leakage resistance R_leak_ representing electrolyte pathways through the membrane. Disruption reduces R_leak_ and increases C_s_, leading to the observed transient in capacitance.

The system demonstrated high sensitivity to both lipophilic and hydrophilic irritants. Irritant application altered the lanolin’s dielectric constant and reduced R′_e_, indicating increased ion mobility. The filter paper absorbed hydrophilic components, enhancing the detection capability across irritant types. Material selection was critical: lanolin, mimicking SC lipids, responded effectively to nonpolar substances, while the hydrophilic filter paper absorbed water-based irritants. The use of PA tape and double-sided tape ensured electrical insulation, mechanical stability, and repeatable measurements.

This layered, biomimetic design enables a reproducible and responsive platform suitable for in vitro irritation assays and supports integration into low-cost biosensor systems.

### 2.5. Capacitive Measurement and Validation of the Cap-S System

Capacitive measurements were performed using the A&A Cap-S module, which features a manufacturer-specified resolution of ±4.0 aF and an intrinsic RMS noise level <2 fF under stable conditions [37]. The system was operated at 1 kHz sampling frequency with a temporal resolution of 0.1 s. Prior to experiments, baseline performance was validated against a commercial precision capacitance meter (GW-INSTEK LCR819, 20 Hz–2 MHz range). Test capacitors (1 pF, 10 pF, 100 pF, 1 nF; tolerance ± 0.1%, Murata) were measured with both instruments, and the Cap-S system showed deviations of less than 1.2% across the range, confirming clinical-grade accuracy for dielectric monitoring.

Baseline stability was further assessed by continuous recording under PBS immersion (no irritant exposure). RMS baseline noise and temperature drift was tested across 20–25 °C. For reproducibility, three independent sensor batches (*n* = 5 per batch) were evaluated with PBS and 5% SDS. Batch-to-batch variation was determined for initial baseline values and for ΔC/Δt under SDS exposure.

These validation steps confirm that the Cap-S system provides reliable, high-resolution capacitance measurements suitable for assessing aSC integrity and irritant-induced barrier disruption.

### 2.6. Preparation and Verification of aSC Coatings on the Sensing Platform

The aSC models were fabricated by applying candidate barrier materials—lanolin, petrolatum, and parafilm—onto mixed cellulose ester (MCE) membranes (pore size: 0.45 μm; nominal thickness: 125 μm; Millipore, Cork, Ireland). For lanolin- and petrolatum-based coatings, 50 μL of molten material was evenly scraped onto a 1 cm^2^ circular cut of MCE membrane and allowed to solidify under ambient laboratory conditions (23 ± 1 °C, 45 ± 5% RH). The uniformity and thickness of the coatings were verified using a digital micrometer (Mitutoyo, ±1 μm accuracy). For lanolin, the coating thickness was consistently 34–42 μm (*n* = 5), while petrolatum yielded 40–46 μm. Parafilm was cut to match the MCE dimensions, pressed onto the membrane surface, and measured to be 120 ± 5 μm thick. All coated membranes were fixed to the sensing electrodes using 3M-9495LE PET-based double-sided (DS) adhesive tape (170 μm nominal thickness).

To ensure reproducibility, all aSC assemblies were prepared in triplicate batches on different days, and batch-to-batch variation in coating thickness was <5%. The measured thickness values were later incorporated into C calibration analysis (see Section 3.1) to correlate barrier layer dimensions with sensor response.

Each candidate material’s coating thickness and barrier effectiveness were verified by applying PBS solution to the sensing surface. A stable capacitance signal over an extended period (>1000 s) indicated successful barrier formation

### 2.7. ATR-IR Spectroscopy

Infrared measurements were recorded using an IRAffinity-1S spectrometer. The crystal probe was cleaned according to the manufacturer’s instructions. The SC sample, obtained by tape-stripping the skin, was mounted on a rigid acrylic backing and secured to the crystal probe using the adjustment knob. ASC samples were prepared as previously described. Each sample was analyzed using ATR-IR spectroscopy, with 10 absorbance scans recorded per spectrum across a range of 4000–400 cm^−1^ at a resolution of 4 cm^−1^, using LabSolution IR 2.3 software. A background spectrum was obtained from a clean crystal with the same backing material used in the sample analysis.

### 2.8. Irritation Test by the Lanolin-Based Model with a Capacitive Sensor (Lanolin + CapS-Based Irritation Assay)

To evaluate the effectiveness of the developed aSC model in identifying skin irritation categories, six commercially available reference substances identified by the OECD for skin corrosion and irritation testing (OECD TG 431, 2004) were selected. These included three substances classified as Category 2 (skin irritants) and three Unclassified substances, according to the UN GHS criteria. All selected substances were liquids. PBS and 5% SDS were used as the negative and positive controls, respectively [38]. Detailed specifications of the substances are provided in Table S1.

The irritation testing protocol confirmed the quality and stability of the aSC model, followed by an extended sample exposure period. The aSC, composed of double-sided tape, filter paper, and lanolin, was freshly prepared for each experiment. Capacitance stability was confirmed by ensuring no significant deviation within the initial 20 s. Then, 50 μL of PBS was applied to cover the two sensing electrode areas for 400 s fully. A lack of significant capacitance change during this blank test verified the integrity of the lanolin barrier. Afterward, the PBS droplet was carefully removed, and the surface was gently blotted with absorbent paper to restore baseline conditions. Subsequently, 50 μL of the test sample was applied to the same area and maintained for at least 400 s for irritation assessment. The maximum capacitance change rate (ΔC/Δt) during this test duration was used as a quantitative indicator of the irritation assay.

Environmental factors—particularly ambient humidity, temperature, and the ionic strength of the contacting medium—can modulate the dielectric properties of both the lanolin aSC layer and the electrode–electrolyte interface, thereby influencing absolute capacitance values and their time-derivatives. To minimize these effects, all measurements were conducted in a controlled laboratory environment (25 ± 1 °C; 50–60% relative humidity), with sensors and coated membranes equilibrated on the bench for ≥10 min prior to testing. We standardized droplet volume (50 µL), exposure duration (7.5 min), and a fixed pre-exposure baseline window, and we report responses as ΔC relative to the immediate pre-exposure baseline. The decision metric ΔC/Δt is computed over a short post-exposure window, which suppresses slow common-mode drift from gradual humidity or temperature fluctuations. Ionic strength was held constant using 1× PBS for all comparative assays; because ionic strength shortens the Debye length and can increase interfacial capacitance, classification experiments used a single, fixed buffer and were bracketed by negative-control PBS runs to monitor day-to-day variation. Practically, these controls (environmental set-points, baseline subtraction, short-window slope estimation, and interleaved controls) rendered environmental contributions small relative to the positive-control (SDS) signals used for classification. For applications requiring operation across wider environmental ranges or diverse matrices, the same framework can be extended with (i) a simple enclosure and on-board temperature/RH logging for post hoc compensation, and (ii) matrix-specific calibration curves (ΔC and ΔC/Δt versus ionic strength) referenced to the PBS control to ensure transferability.

### 2.9. Data Processing and Definition of Parameters

The raw capacitance signal was recorded continuously at a temporal resolution of 0.1 s using the A&A Cap-S module. Two parameters were extracted to characterize the sensor response: the absolute capacitance change (C) and the capacitance change rate (ΔC/Δt).

C (fF or pF) was defined as the difference in capacitance between the baseline (prior to chemical exposure) and the subsequent signal during exposure. All reported ΔC values are presented after subtraction of the air-exposure response, ensuring that only solvent- or irritant-induced changes are considered.

ΔC/Δt (fF/s) was defined as the first derivative of capacitance with respect to time, reflecting the rate of barrier perturbation under chemical exposure. To avoid confusion, we consistently use lowercase t to denote time, whereas uppercase T is reserved for temperature. The ΔC/Δt parameter was calculated from the linear portion of the capacitance–time curve between 60 s and 450 s after application of the test compound, a window selected to exclude initial transients (liquid spreading) and capture the steady-state penetration phase.

A ‘stable C plateau’ refers to the point at which the capacitance change reached a quasi-equilibrium after exposure, typically observed following the transient ΔC/Δt phase. This terminology clarifies that it is the capacitance change that stabilizes, rather than the absolute capacitance baseline.

The ΔC/Δt metric was proposed as a quantitative indicator of irritant-induced barrier disruption because it emphasizes the kinetics of dielectric property alteration in the artificial stratum corneum layer, rather than relying solely on the end-point magnitude of C.

### 2.10. Statistical Analysis

Calculations and statistical analyses were performed using Microsoft^®^ Excel^®^. Data are presented as mean ± standard deviation (SD). Statistical significance was assessed using a *t*-test, with the Bonferroni correction applied for multiple comparisons. Differences were considered significant at *p* < 0.01.

## 3. Results

### 3.1. Impact of Dielectric Spacer Thickness on Capacitance Response and Calibration for the Sensing Platform

To evaluate the effect of dielectric spacer thickness on capacitive sensor response, we performed calibration measurements using PET-based double-sided (DS) tapes with nominal thicknesses of 0 μm (no spacer), 30 μm, 130 μm, and 330 μm. Each DS tape was placed between the electrodes as a dielectric spacer, and the change in capacitance (ΔC) was recorded over time during electrolyte application and removal.

**Figure 2:**
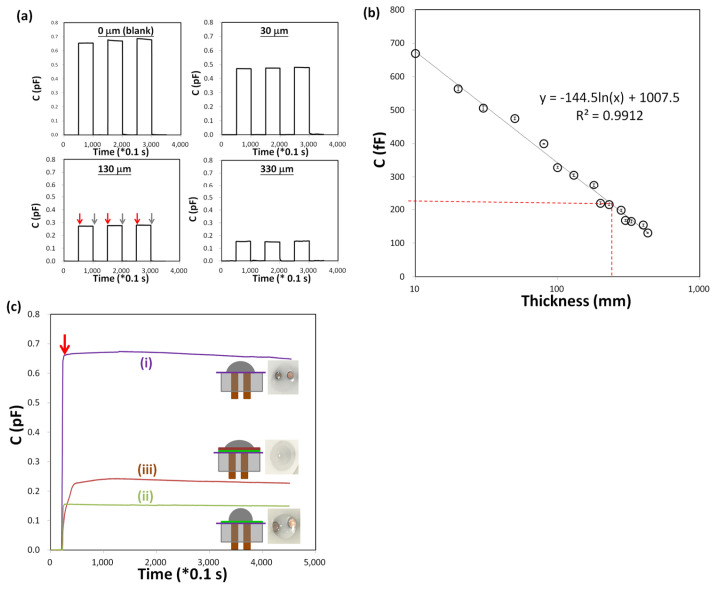
Capacitive sensor responses to varying dielectric spacer thicknesses and sensing layer configurations. (**a**) Triple-repeat real-time response signal of the change in capacitance (C) over time for different spacer thicknesses: 0 μm (no spacer), 30 μm, 130 μm, and 330 μm. The red and grey arrows indicate, respectively, the time points at which the electrolyte drips onto and is removed from the sensing area. (**b**) Calibration curve of C vs. spacer thickness, with a logarithmic fit (C = −144.5 ln(x) + 1007.5, R^2^ = 0.9912). A horizontal red line at C = 220 fF marks the working point used in the study for constructing an artificial stratum corneum using 3M-9495LE double-sided tape (thickness 170 μm). The Cap-S module employed an offset capacitance of ±4.025 pF. (**c**) Capacitive responses of the sensor to PBS electrolyte on various configuration of sensing layer. (i) basic insulation (BI); (ii) DS tape/BI; (iii) mixed cellulose ester filter paper (MCE/DS/BI). The red arrow indicates when the electrolyte is dripping on the sensing surface. Cartoon-style convex display configuration structure side view. The photo shows the top view after the electrolyte drops.

The experimental ΔC responses shown in Figure 2 are consistent with the equivalent circuit representation, where the interfacial membrane capacitance (C_s_) accounts for the baseline signal, and the barrier-associated elements (C_s_ and R_leak_) correspond to the observed capacitance decrease with increasing spacer thickness, thereby validating the applicability of Equations (1)–(3) to interpret the measured data.

Figure 2a shows four typical histograms of C (change in capacitance, pF) over time for various thicknesses, revealing that C decreases as thickness increases, with distributions narrowing at larger thicknesses. It shows narrowing distributions at larger thicknesses (e.g., 330 μm vs. 0 μm), potentially indicating reduced measurement variability or noise. This could imply better stability at higher thicknesses, possibly due to less sensitivity to external factors, which is crucial for reliable irritation assays.

Figure 2b provides a calibration curve, demonstrating a logarithmic decrease in ΔC with increasing thickness, fitted with the equation C = −144.5 ln(x) + 1007.5, where x is thickness in μm and C is in femtofarads (fF), with a strong fit (R^2^ = 0.9912). The strong fit suggests the model is reliable for calibration, but the deviation at the tape point indicates material effects must be considered. The decrease in ΔC with increasing thickness aligns with capacitive sensor theory, where capacitance decreases with greater distance between plates (C = εA/d, where d is distance). The logarithmic fit suggests a non-linear, diminishing effect, confirming robustness. The blank condition (0 μm) shows a high C, which has the most considerable capacitance change value (669 fF) because there is only a skinny insulating layer between the electrode pair and no additional thickness-controlled spacer (double-sided tape).

The highlighted point at 170 μm with DS tape (3M-9495LE), labeled at 170 μm with C ≈ 220 fF, suggests a discrepancy. Calculating for 170 μm: C = 264.14 fF. This is higher than 220 fF, indicating the actual measured C for the tape may differ, likely due to material properties (PET composition, tape structure) beyond just thickness. This underscores the need for material-specific calibrations in sensor applications.

These results imply that thinner DS tapes provide wider detection range (from C = 0 fF to working point) due to more capacitive sensing margin. More detailed explanations will be in the following section (Figure 2c). The working point at C = 220 fF, corresponding to ~255 μm, is used to adhere the sensing layer for constructing an aSC in this study. Although using a thinner DS tape (i.e., lower thickness adhesive) could further extend the measurement range and potentially improve response sensitivity, other factors were prioritized. In particular, very thin DS tapes are often more difficult to handle during assembly and can be less available in standard laboratory supply channels. Additionally, cost-effectiveness was considered, as readily available mid-thickness DS tapes provide adequate performance at a lower procurement cost. The detection limit of the capacitive sensing module (A&A Cap-S) used in this study is already well above the requirement for our target application, so the choice of a slightly thicker, commercially common DS tape does not significantly affect measurement performance. For applications with different requirements, the working point and measurement range can be adjusted using the calibration curve in Figure 2b.

### 3.2. ATR-IR Spectral Analysis of Candidate Materials for aSC

**Figure 3:**
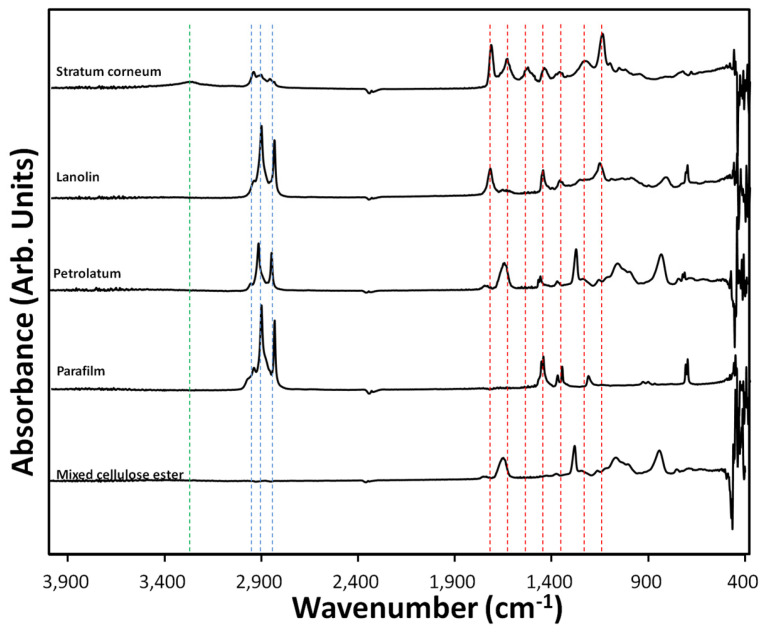
ART-IR spectra of skin stratum corneum (SC), artificial SC membranes (lanolin, petrolatum, parafilm), and mixed cellulose ester filter paper. Green broken line indicates a peak at 3300 cm^−1^ (suggests O-H or N-H stretching); three blue broken lines indicate peaks at 2960 cm^−1^, 2920 cm^−1^ and 2850 cm^−1^ (C-H stretching); seven red broken lines indicate peaks at 1740 cm^−1^ (C=O stretching), 1650 cm^−1^ (amide I, C=O stretching), 1550 cm^−1^ (amide II, N-H bend and C-N stretching), 1460 cm^−1^ (C-H bending), 1380 cm^−1^ (C-H bending), and 1250 cm^−1^ (possibly C-N stretching or amide III), and 1170 cm^−1^ (C-O stretching). See text for details.

ATR-FTIR spectroscopy was employed to investigate the molecular compositions of human SC and candidate materials—lanolin, petrolatum, Parafilm, and mixed cellulose ester (MCE) filter—used for constructing an aSC model (Figure 3). The objective was to identify key functional groups associated with barrier function, enabling material selection based on spectral similarity to native SC.

The SC spectrum exhibited characteristic features of lipids and proteins. A broad absorption at ~3300 cm^−1^ corresponded to O–H or N–H stretching, attributed to water and amide groups. Strong aliphatic C–H stretching bands appeared at 2920 and 2850 cm^−1^, while amide I and II bands were identified at 1650 and 1550 cm^−1^, respectively, indicating protein content. Additional bands at 1450 and 1250 cm^−1^ were assigned to C–H bending and C–N stretching, consistent with its keratin-rich composition [39,40,41].

Lanolin, a wool-derived wax, showed broad O–H stretching near 3300 cm^−1^, aliphatic C–H stretching at 2920 and 2850 cm^−1^, and a strong ester C=O peak at 1740 cm^−1^. Further peaks at 1460 and 1170 cm^−1^ (C–H bending and C–O stretching) confirmed the presence of complex lipid esters. The peak at 720 cm^−1^, indicative of long-chain alkyl groups, reflected its similarity to SC lipid components.

Petrolatum displayed a simpler hydrocarbon profile, with C–H stretching and bending bands at 2920, 2850, 1460, and 1375 cm^−1^, and a C–H rocking band at 720 cm^−1^. Absence of O–H or C=O signals confirmed its non-polar, saturated hydrocarbon nature. Parafilm exhibited nearly identical features, consistent with its paraffin-based composition.

The MCE filter spectrum included weak O–H stretching, C–H stretching at 2920 and 2850 cm^−1^, and a C=O stretch at 1740 cm^−1^, suggesting esterification of cellulose. Additional peaks at 1375, 1230, and 1050 cm^−1^ were assigned to C–O and C–O–C stretching, indicating glycosidic linkages and ester functionalities.

These findings support the selection of lanolin as a primary aSC component due to its spectral resemblance to native SC in both lipid and protein domains. Petrolatum and Parafilm serve as hydrophobic barriers, while MCE contributes mechanical support and fluid absorption. The comparative spectral analysis provides a rational basis for material selection in constructing biomimetic barriers for irritation assays and skin-mimicking biosensor platforms.

Lanolin was selected as the primary aSC material because its lipid composition closely mirrors that of the native SC. It contains a complex mixture of sterol esters, long-chain fatty acids, and aliphatic alcohols, which resemble the amphiphilic lipid matrix of human SC. This compositional similarity supports its ability to reproduce barrier integrity and dielectric changes under irritant exposure, thereby providing a mechanistic rationale for the observed ΔC/Δt responses. Such mimicry strengthens the use of lanolin-based coatings as a functional surrogate for natural SC in capacitive irritation assays.

### 3.3. Evaluation of Candidates for aSC: Capacitive Response and Material Suitability

**Figure 4:**
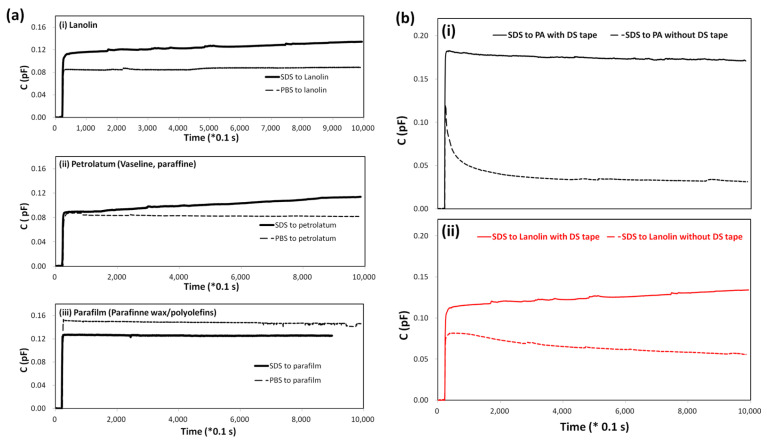
Capacitive responses of candidate lipid coatings and fixation methods for artificial stratum corneum construction. (**a**) Capacitive responses of various lipid-mixed coatings to negative and positive control compounds for potential artificial stratum corneum candidates. (**i**) Lanolin; (**ii**) Petrolatum; (**iii**) Parafilm. PBS and 5% SDS were used as negative and positive controls, respectively. A 50 μL sample was applied to the coated sensing surface at t = 20 s. Capacitance was measured using the Cap-S system. (**b**) The reliable response depended on the double-sided tape utilized. SDS contact with Polyacetate (PA) film (**i**) and lanolin/mixed cellulose ester filter paper (**ii**). Solid and broken lines are indicated as fixed with double-sided and cover tape, respectively.

A preliminary evaluation of candidate materials for aSC construction was conducted based on two criteria: the ability to maintain stable capacitance under neutral conditions (PBS) and to exhibit a gradual increase upon exposure to a surfactant (SDS). This assessment was guided by the data presented in Figure 4a, in which lanolin, petrolatum, and parafilm were compared.

After applying the sensing overlay (t = 20 s), variations in the initial capacitive response (ranging from 0.15 to 0.8 pF) were observed. Inconsistencies likely influenced these differences in layer thickness and configuration during preparation. While such variability could be addressed through refined fabrication methods in future studies, it was not considered critical to identify C changes following SDS exposure

All three materials demonstrated stable responses to PBS, confirming their reliability as coating layers. However, no significant change in capacitance was observed in parafilm following SDS application, suggesting its strong barrier integrity but limited suitability for irritation detection. In contrast, lanolin and petrolatum displayed more pronounced responses to SDS, each showing a comparable ΔC/Δt value of approximately 2 fF/s, indicating adequate sensitivity to barrier disruption.

These results suggest that parafilm, due to its high resistance to SDS, may be less appropriate for mimicking SC permeability in this context. Lanolin and petrolatum, by contrast, offered greater sensitivity to surfactant-induced changes and are therefore more promising for use in aSC models. When considered alongside previous functional group analyses, lanolin was identified as the most suitable material, offering a favorable balance of barrier function and responsiveness for use in irritation assays.

### 3.4. Evaluation of Fixation Methods for Sensing Layers in Capacitive Sensors: Double-Sided (DS) vs. Cover Tape

The effectiveness and reliability of sensing layer fixation using double-sided (DS) and single-sided (cover) tapes were evaluated, as presented in Figure 4b. This investigation used a lanolin/CE filter paper layer as the sensing element. Two fixation methods were compared: DS tape, which features adhesive on both sides, and cover tape, which utilizes single-sided adhesion.

For the PA film, a higher initial capacitance change (C = 0.17 pF) was recorded when DS tape was applied, compared to 0.12 pF with cover tape. This result suggests that stronger initial contact was achieved with DS tape, likely due to improved surface conformity and reduced lateral movement. A minimal signal decay was observed over time with DS tape, while the cover tape configuration converged to 0.004 pF, indicating a decline in measurement stability over prolonged durations.

In the case of lanolin-coated MCE filter paper, the sensor stabilized at 0.115 pF with DS tape and 0.08 pF with cover tape. These findings underscore the importance of robust fixation for porous or uneven materials, where DS tape provided a more stable sensing interface. Contrary to expectations, the performance of the sensing layer was significantly influenced by the tape type, reinforcing the role of mechanical anchoring in maintaining signal integrity.

The superior adhesion provided by DS tape was likely responsible for minimizing mechanical noise and mitigating measurement variability. A more uniform interface was presumably formed, reducing voids or discontinuities that could influence dielectric behavior. These effects were particularly evident in Figure 4b, where DS tape maintained signal stability at 0.11 pF, while the signal dropped to 0.06 pF under the cover tape condition. Since capacitive sensing relies on detecting variations in distance or dielectric properties, a secure and consistent interface was deemed critical.

Additionally, the use of single-sided tape was found to introduce significant variability. The test solution penetrated through the edge of the tape, reaching the lower filter layer and compromising reproducibility. This defect further limits the suitability of cover tape for precision capacitive measurements.

To further evaluate the influence of fixation methods on measurement stability, we compared responses obtained using DS tape versus cover tape (Figure 4b). The DS tape consistently produced more stable and reproducible capacitance signals, with reduced baseline drift and lower variability across repeated measurements. In contrast, cover tape fixation exhibited irregular signal fluctuations, indicating weaker adhesion and less uniform contact at the sensing interface. These results confirm that DS tape fixation enhances the robustness of the aSC assembly, providing a more reliable platform for irritation assays.

Overall, DS tape provided superior fixation, particularly for irregular or absorbent substrates. Its application enhanced signal reliability and long-term stability, making it more appropriate for sensitive assays such as skin irritation testing or environmental sensing. Based on these findings, DS tape is recommended for future use in capacitive sensor configurations requiring consistent and robust layer attachment.

### 3.5. Representative Capacitive Responses of Lanolin-Coating to Surfactant-Induced Barrier Disruption

To ensure that the observed capacitance responses reflected barrier perturbation rather than instrumental or environmental artifacts, we systematically evaluated baseline stability, reproducibility, and robustness of the capacitive sensing system. The A&A Cap-S module provides a specified resolution of ±4.0 aF with intrinsic RMS noise <2 fF under stable conditions [37]. Control measurements in PBS (no stimulus) were consistent with this specification, showing RMS baseline fluctuations of 1.8 ± 0.3 fF over 600 s (*n* = 10). Temperature drift analysis between 20 ± 0.5 °C and 25 ± 0.5 °C revealed only a 0.012 ± 0.004 fF/°C shift, negligible compared to C values exceeding 200 fF under chemical exposure. Batch-to-batch reproducibility across three independently fabricated sensor sets (*n* = 5 each) showed <0.7% variation in baseline capacitance and <3.1% variation in ΔC/Δt under SDS, confirming fabrication consistency. Robustness was further demonstrated by intra-day, inter-day, and inter-device comparisons. Intra-day repeatability (*n* = 5) produced coefficients of variation (CVs) of 2.3% for PBS and 2.5% for SDS. Inter-day testing across three consecutive days gave CVs of 2.8% (PBS) and 3.0% (SDS). Inter-device variability across three independently fabricated sensors yielded <3.5% variation in ΔC/Δt under SDS exposure. Collectively, these results establish the system’s stability and reproducibility across time, batches, and devices, ensuring that capacitance changes arise from true barrier-disrupting interactions rather than noise or drift.

**Figure 5:**
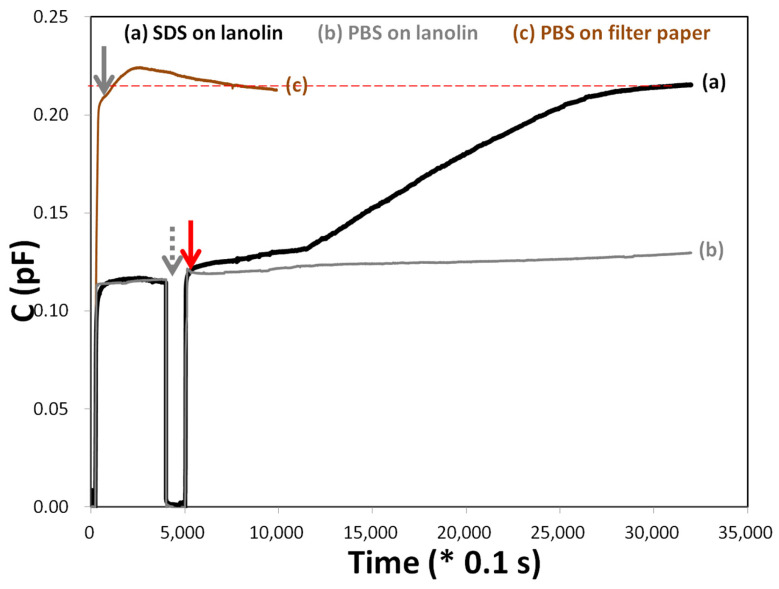
Representative capacitive response of lanolin-coated mixed cellulose ester filter paper upon exposure to SDS. (a) 10% SDS; (b) PBS (pH 7.4); (c) PBS applied to filter paper without lanolin coating. The gray arrow indicates PBS being added to the sensing surface, while the gray dashed arrow marks its removal. The red arrow signifies the addition of the sample solution (e.g., 10% SDS) to the sensing surface. The red thin dashed line represents the steady-state response as the electrolyte fully permeates the sensor surface. The slope ΔC/Δt, used as the quantitative indicator of barrier disruption, is directly calculated from these raw curves. The comparison of responses to PBS (non-irritant) and SDS (irritant) provides a clear visual separation, directly demonstrating the sensor’s ability to distinguish irritant from non-irritant exposures.

In Figure 5, the capacitive response of the lanolin-coated layer to SDS was characterized. A sharp increase in C was observed in the reference layer (brown line), beginning at t = 20 s and reaching approximately 0.22 pF by t = 500 s. This response was attributed to the uncoated MCE filter paper, which allowed rapid penetration or interaction with PBS, resulting in a higher capacitance. In contrast, the negative control (gray line) exhibited a minor decrease in C following PBS removal, maintaining a relatively stable capacitance of approximately 0.11 pF throughout the experiment. These results confirmed that PBS was effectively repelled by the lanolin-coated filter, supporting lanolin’s role as a hydrophobic barrier against water-based solutions.

The black line in the figure remained near 1.1 pF until approximately t = 4000 s. At t ≈ 5000 s, a 10% SDS solution was introduced, as indicated by the red arrow. Following this introduction, a slow transient increase in capacitance was observed between 500 s and 1200 s, followed by a gradual rise. From t ≈ 1200 s to t = 3500 s, C increased steadily, reaching approximately 0.20 pF by the end of the measurement. This gradual change suggested that the lanolin barrier was progressively compromised by SDS, allowing eventual complete permeation. The rate of capacitance change (ΔC/Δt) following 500 s was adopted as a quantitative indicator in the subsequent analysis.

The hydrophobicity of lanolin effectively prevented penetration by PBS, leading to negligible capacitance changes. This behavior was consistent with previously reported applications of lanolin in waterproofing and corrosion prevention [42]. In contrast, SDS—a surfactant—was found to disrupt the lanolin barrier by reducing surface tension, thereby facilitating the interaction of water and ions with the underlying filter paper and resulting in a measurable increase in capacitance. This mechanism aligned with SDS’s known ability to enhance the permeability of hydrophilic substances through hydrophobic barriers [43]. Rapid interaction with PBS was observed in the uncoated Advantec filter, leading to a capacitance increase that exceeded steady-state values. This behavior was likely due to the absence of a hydrophobic barrier and the distinct surface properties of the material.

These findings demonstrated that lanolin-coated papers could be applied in capacitive sensing scenarios where selective permeability is required. They effectively block water-based solutions while allowing gradual, surfactant-assisted penetration. Furthermore, the interaction between surfactants and lanolin barriers may inform the design of future controlled-release or drug delivery systems. Furthermore, these observations are consistent with the equivalent circuit in Figure 1c, where the artificial stratum corneum behaves as a dielectric barrier in series with the electrical double layer. Irritant-induced barrier disruption increases the effective dielectric constant and decreases the leakage resistance, producing the observed transient increase in capacitance. The rate ΔC/Δt therefore provides a quantitative indicator of the kinetics of membrane compromise.

In summary, the lanolin-coated MCE filter paper served as an effective barrier against PBS, preventing significant capacitance shifts, and allowing a gradual capacitance increase in response to SDS exposure. In contrast, the uncoated filter paper allowed PBS to penetrate it immediately. These results reinforced the role of lanolin in regulating permeability and suggested promising applications in sensor development, barrier material evaluation, and skin irritation assays.

### 3.6. Lanolin-Based aSC and Capacitive Sensing for In Vitro Skin Irritation Classification

**Figure 6:**
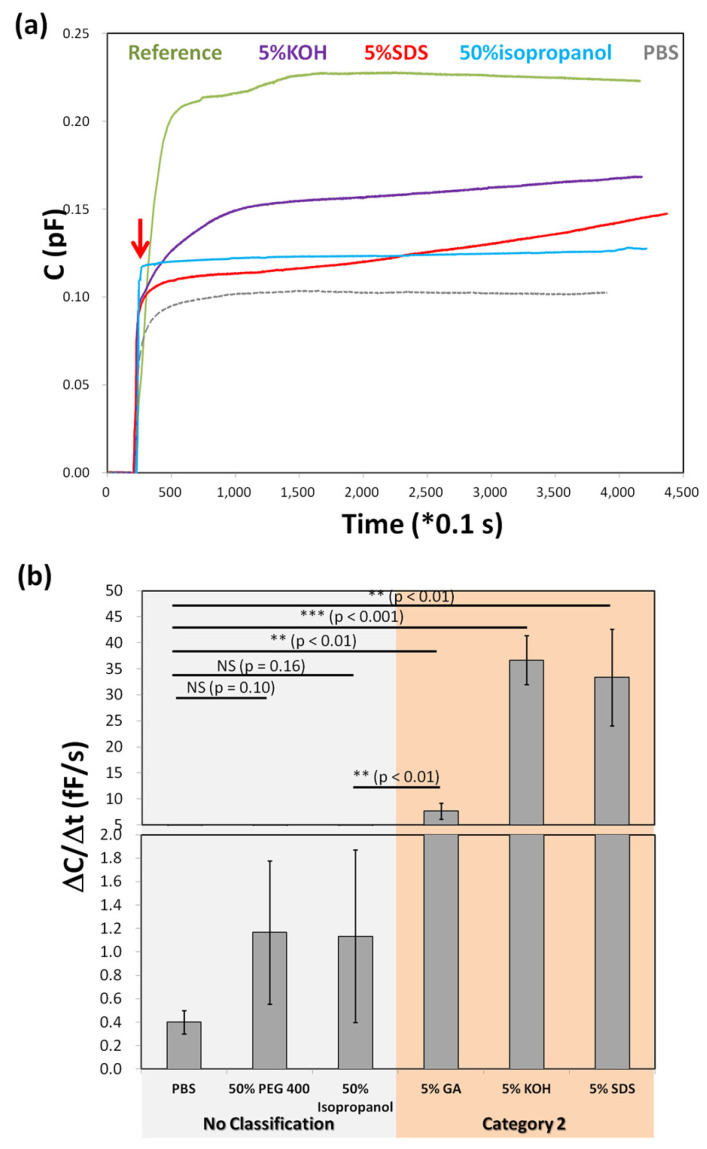
Irritation assay of irritants by the lanolin-based model with a capacitive sensor. (**a**): Real-time capacitive responses of lanolin-based artificial stratum corneum (aSC) to various compounds with irritation levels. PBS and 5% SDS are respectively OECD-approved negative and positive controls used in the irritation assay. 5% KOH and isopropanol are defined as Category 2 and No Classification compounds, respectively. The red line arrow indicates that the sample was dropped on the surface of the aSC. (**b**): In vitro irritation assay of compounds grouped to No Classification and Category 2. No Classification compounds: PBS, PEG 400, and isopropenol; Category 2 compounds: GA, KOH, and SDS. *** *p* < 0.001; ** *p* < 0.01; * *p* < 0.1; NS *p* ≥ 0.1, NS: not significant. The asterisk * indicates a significant difference from the PBS control in the Student’s *t*-test (*n* = 3).

Figure 6a presents the real-time capacitive responses of the lanolin-based aSC model upon exposure to different compounds over a 7.5-min period. These measurements reflect barrier integrity changes, allowing non-animal assessment of skin irritation potential. When PBS was applied without lanolin (bare filter), capacitance increased sharply and plateaued near 0.22 pF, indicating rapid barrier loss. In contrast, the lanolin-coated model exposed to known irritants—5% SDS and 5% KOH, both classified as Category 2—exhibited a steady increase in capacitance, reaching ~0.15 pF and ~0.12 pF, respectively, by 400 s. These profiles suggest gradual barrier disruption typical of irritants.

For non-irritants, such as PBS and 50% isopropanol, capacitance remained stable, below 0.10 pF. Isopropanol showed a mild increase, reflecting its known potential for cumulative barrier damage, though not sufficient to trigger irritant classification under short exposure. Interestingly, 5% KOH generated a higher C than SDS at the same concentration, possibly due to its strong basicity and enhanced lipid disruption capability. However, SDS is typically more irritating under prolonged exposure, suggesting that time-dependent effects warrant further study.

Figure 6b quantifies these responses using ΔC/Δt (change in capacitance over time) as a dynamic metric for evaluating irritant potential. The “No Classification” group—comprising PBS, 50% PEG 400, and 50% isopropanol—yielded ΔC/Δt values ranging from 0.4 to 1.3 fF/s. These values were not statistically different from the PBS control, supporting their classification as non-irritants. Conversely, the “Category 2” group—5% GA, 5% SDS, and 5% KOH—displayed significantly higher ΔC/Δt values (8–40 fF/s, *p* < 0.01), consistent with their known irritant properties.

The aSC model mimics the skin’s outermost barrier, and the capacitive changes indicate alterations in its electrical properties upon chemical exposure. This approach aligns with prior research using capacitance imaging and sensor-based hydration models [35]. Although isopropanol produced a statistically significant increase compared to PBS, it remained within the non-irritant threshold [44], illustrating the value of using defined cutoffs (e.g., 3.5 fF/s) for classification.

This model offers a promising, ethically responsible alternative to animal and cell-based irritation assays. Its ability to rapidly distinguish irritants from non-irritants through quantitative capacitance metrics demonstrates potential for regulatory screening, especially in the cosmetics and pharmaceutical sectors.

The current classification relied on a binary system (“No Classification” vs. “Category 2”) based on EU CLP guidelines. However, finer gradations exist in systems like the UN GHS, which includes Category 1 (corrosives) and Category 3 (mild irritants). The present method successfully identified Category 2 irritants, but variability within the No Classification group (e.g., 1.3 fF/s for isopropanol vs. 0.4 fF/s for PBS) suggests sensitivity to subtle differences. Expanding the dataset to include Category 1 and 3 substances would allow for refinement of threshold definitions and enhance model precision.

Notably, even non-irritants like 50% PEG 400 exhibited measurable responses, although not statistically significant [45]. This supports the model’s capacity to detect low-grade or subclinical effects [44], which could prove valuable in distinguishing mild irritants—such as essential oils or formulation excipients—from truly inert materials. Such granularity is useful in screening products for dermal biocompatibility, especially in medical device and cosmetic applications [46].

Despite its strengths, this study evaluated only six compounds, limiting generalizability. To establish regulatory acceptance, further validation is necessary, including testing with broader chemical classes, incorporation of Category 1 and 3 references, and inter-laboratory reproducibility studies. Comparison with other artificial skin platforms and in vivo data would also be important for establishing physiological relevance [47].

The capacitance change rate (ΔC/Δt) was validated as a reproducible quantitative parameter (CV < 3.5%) that effectively discriminated irritants (SDS) from non-irritants (PBS, PEG400). Although direct benchmarking against OECD TG 439 or reconstructed human epidermis (RhE) models was not included in this proof-of-concept, the observed trends are consistent with established irritation assays. These results highlight ΔC/Δt as a mechanistically grounded, indicator-free endpoint with strong potential for integration into regulatory non-animal testing frameworks.

The lanolin-based aSC capacitive assay demonstrates a clear ability to differentiate irritants from non-irritants via rapid, quantitative sensing of barrier disruption. While additional data are needed to refine classification thresholds and improve coverage across irritation categories, this approach holds strong promise as an efficient, ethical, and scalable alternative to traditional skin irritation tests.

## 4. Discussion

A comparative evaluation of five in vitro skin irritation models revealed clear differences in complexity, cost, duration, and biological relevance (Table S2). The lanolin + Cap-S-based model, central to this study, employs a capacitive sensor (A&A Cap-S) to assess barrier disruption through real-time monitoring of capacitance changes (ΔC/Δt, in fF/s). Measurements are completed within one hour, offering a rapid and low-cost solution for irritation screening without the need for complex instrumentation.

In contrast, reconstructed human epidermis (RHE)-based models (e.g., EpiSkin^®^, EpiDerm^®^) evaluate irritation through keratinocyte viability using MTT assays. While biologically relevant, these models are labor-intensive, expensive, and time-consuming. Synthetic membrane-based models (e.g., Irritection Dermal^®^) detect dye release through protein membranes and typically require 24–48 h. Similarly, corrosion-specific models (e.g., Corrositex^®^) monitor chemical penetration through collagen gels using spectrophotometric readouts, though they target corrosivity rather than irritation. Drug delivery models (e.g., Strat-M^®^, Nucleopore^®^) focus on compound permeation and rely on HPLC for analysis, limiting their application in irritation screening.

While all models are limited by their synthetic or simplified nature, the lanolin + Cap-S model demonstrates superior practicality and direct alignment with irritation endpoints. Although RHE-based models offer increased biological relevance, their reliance on metabolic viability may underrepresent direct barrier damage. In contrast, the capacitive model targets barrier integrity as a primary irritation marker, yielding a mechanistically relevant and quantitative output.

The model’s simplicity, affordability, and rapid data acquisition make it ideal for early-stage screening or routine testing [48]. Its reduced biological complexity is a trade-off, but one that may be acceptable depending on the study objectives. For high-throughput needs or formulation triage, the lanolin + Cap-S model offers significant operational benefits.

However, like all current models, it cannot fully replicate the multilayered architecture and immune interactions of living skin. RHE systems incorporate keratinocytes but still fall short in mimicking immune responses and dynamic in vivo behaviors. Future development should focus on integrating synthetic systems with reconstructed tissues or ex vivo platforms. Hybrid systems combining immune cells or structural lipids may bridge the gap between practicality and biological fidelity.

In acknowledging the limitations of this study, it should be noted that volatile or non-polar compounds may interfere with capacitance responses due to their limited interaction with the lanolin-based aSC layer. In addition, potential batch-to-batch variability in lanolin quality could affect barrier consistency, and direct in vivo or in vitro correlation data are not yet available. These limitations highlight areas for future validation and refinement to strengthen the translational relevance of this platform.

Looking ahead, the sensing platform can be readily tuned to model a broader spectrum of skin types, barrier states, and irritation scenarios. By adjusting the dielectric spacer thickness and material properties, the adequate sensing depth and baseline capacitance can be tailored to mimic anatomical variations in stratum corneum thickness or hydration levels. Furthermore, the lipid composition of the artificial stratum corneum (aSC) can be modified to replicate altered barrier conditions, such as those found in aged skin, atopic dermatitis, or chemically compromised epidermis. Chronic irritation models could be developed by implementing repeated low-dose exposure protocols to monitor barrier degradation and recovery dynamics over time. These adaptations, combined with the calibration framework established in this work, would enable quantitative comparison between diverse skin analogues and extend the platform’s applicability to long-term safety assessments in cosmetic, pharmaceutical, and dermatological research.

Beyond its current single-sensor configuration, the lanolin-based artificial stratum corneum capacitive platform is readily adaptable to multiplexed or automated high-throughput screening formats. The compact electrode geometry and low sample volume requirements make it compatible with multi-well plate layouts or microfluidic chamber arrays, where multiple sensing units can operate in parallel. By integrating with commercially available multi-channel capacitance measurement systems, ΔC/Δt responses from numerous sites could be recorded simultaneously, allowing rapid screening of large compound libraries. The indicator-free nature of the sensing process further facilitates automation, as it removes the need for reagent addition or wash steps, reducing assay time and minimizing operator intervention. Such scalability positions the platform as a promising tool for early-stage toxicological evaluation, cosmetic formulation testing, and pharmaceutical pre-screening, particularly in settings where throughput and reproducibility are critical.

The lanolin-based aSC capacitive biosensing platform demonstrates clear potential for integration into regulatory non-animal testing frameworks (e.g., OECD TG 439) and consumer safety evaluations, offering a rapid, label-free approach to irritant classification. Its modular architecture readily supports multiplexing and automation, enabling scalability for high-throughput industrial use. Collectively, the lanolin + Cap-S irritation model represents a practical, ethical, and scalable alternative to animal-based skin irritation assays. By focusing on barrier disruption as the primary endpoint and combining this with operational efficiency, the system is well positioned for adoption in early-stage screening within cosmetic, pharmaceutical, and regulatory pipelines.

## 5. Conclusions

This study presented a lanolin-based aSC model integrated with a capacitive sensing system for in vitro skin irritation testing. The platform detects irritant-induced changes by monitoring shifts in interfacial dielectric properties, enabling indicator-free and real-time evaluation of barrier disruption. Among the tested materials—lanolin, petrolatum, and Parafilm—lanolin emerged as the most suitable for constructing the sensing layer due to its hydrophobicity, stable baseline in PBS, and responsive capacitance increase upon exposure to SDS. These features closely resemble the functional characteristics of human SC, maintaining stability under neutral conditions while responding to irritants.

Quantitative assessment was achieved through the rate of capacitance change (ΔC/Δt), which distinguished irritants like SDS and KOH from non-irritants such as PBS and isopropanol. The sensitivity and reproducibility of the system were further optimized by evaluating layer thickness and configuration.

The proposed capacitive platform operates without frequency sweeping, dyes, or complex instrumentation, enhancing accessibility and reducing system complexity. By capturing dielectric changes during compound permeation, the sensor provides early-stage irritation screening aligned with ethical and regulatory needs. This lanolin-based aSC platform aligns with the **3Rs principle** by reducing reliance on animal and cellular models for irritation testing. From a regulatory standpoint, its classification potential complements established frameworks such as OECD TG 439, supporting its role as a scientifically robust alternative method. Furthermore, the platform’s simplicity and adaptability highlight its industrial relevance, offering a practical route for high-throughput screening in pharmaceutical, cosmetic, and chemical safety evaluations.

Beyond irritation assays, the system holds potential for applications in cosmetic safety, pharmaceutical development, and chemical screening. Future adaptations may include permeability profiling or transdermal delivery assessments. In summary, the lanolin-based aSC model with capacitive sensing serves as a scalable, sensitive, and ethical tool for evaluating skin interactions in diverse biomedical contexts.

## Data Availability

The original contributions presented in this study are included in the article/Supplementary Material. Further inquiries can be directed to the corresponding author(s).

## Supplementary Materials

The following supporting information can be downloaded at: https://www.mdpi.com/article/10.3390/bios15090564/s1, Table S1. List of 6 test substances with their CAS number, physical state, chemical state, irritation in vivo score and in vivo UN GHS classification. Table S2. Comparison of the lanolin + Cap-S-based model with similar core technologies and applications.
