# Supplementary material for: Capacitive Biosensing of Skin Irritants Using a Lanolin-Based Artificial Stratum Corneum Model
Source: Biosensors (Basel). 2025 Aug 28;15(9):564. doi: 10.3390/bios15090564 (PMC12467698; doi:10.3390/bios15090564)
Supplement: Supplementary file 1 [file biosensors-15-00564-s001.zip › biosensors-3806988-supplementary.pdf]

biosensors

Supplementary Materials

Capacitive Biosensing of Skin Irritants Using a Lanolin-Based Artificial Stratum Corneum Model

Chung-Ting Cheng [1,2], Yi Kung [3], Hung-Yu Chen [1], Kuang-Hua Chang [1,4], Richie L. C. Chen [1] and Tzong-Jih Cheng [1,*]

1 Department of Biomechatronics Engineering, College of Bio-Resources and Agriculture, National Taiwan University, Taipei 106319, Taiwan; b11b02013@ntu.edu.tw (C.-T.C.); d13631001@ntu.edu.tw (H.-Y.C.); kuanghua@ntu.edu.tw (K.-H.C.); rlcchen@ntu.edu.tw (R.L.C.C.)
2 Department of Biochemical Science & Technology, College of Life Science, National Taiwan University, Taipei 106319, Taiwan
3 Department of Biomechatronic Engineering, College of Science and Engineering, National Chiayi University, Chiayi 60004, Taiwan; yikung@mail.ncyu.edu.tw;
4 Experimental Farm, College of Bio-Resources and Agriculture, National Taiwan University, Taipei 106319, Taiwan
* Correspondence: tzongjih@ntu.edu.tw

Table S1. List of 6 test substances with their CAS number, physical state, chemical state, irritation in vivo score and in vivo UN GHS classification.

No.	Substance	CAS#	Physical state	Chemical class	Irritation score *	In vivo UN GHS class
**1**	Sodium dodecyl sulfate (SDS, 5%)	151-21-3	liquid	Soap/ surfactant	4	Category 2
**2**	Potassium hydroxide (KOH, 5% aq.)	1310-58-3	liquid	Basic inorganic	3	Category 2
**3**	Glutaraldehyde (GA, 5%)	111-30-8	liquid	Acidic organic	2.125[#]	Category 2
**4**	Isopropanol (50%)	67-63-01	liquid	Neutral organic	0.3	No Classification
**5**	Polyethylene glycol 400 (PEG-400, 50%)	25322-68-3	liquid	Neutral organic	0	No Classification
**6**	Phosphate-buffered saline (PBS, pH7.3)	-	liquid	Neutral inorganic	0	No Classification

* In vivo score in accordance with the OECD TG 404 (4).

[#] DECOS and NEG Basis for an Occupational Standard Glutaraldehyde. ISBN 91–7045–439–6.

Table S2. Comparison of the lanolin + Cap-S-based model with similar core technologies and applications.

Aspect	Lanolin + Cap-S-based Irritation Model (this study)	RHE-based Irritation models (e.g., EpiSkin, EpiDerm)	Synthetic Membrane-based irritation Model (e.g., Irritection Dermal)	Synthetic Membrane-based Corrosion Model (e.g., Corrositex)	Lanolin-Based Drug Delivery Model - percutaneous absorption assay (/Strat-M® and Nucleopore®)
**Primary use**	Irritation	Irritation	Irritation	Corrosion	Drug delivery
**Principle**	Barrier disruption	Cell availability	Barrier disruption & dye release	Penetration	Permeation
**Materials**	Lanolin	Keratinocyte	Keratin & collagen (membrane)	Collagen gel (membrane)	Lanolin
**Measurement**	Changes in Capacitance (DC/Dt in fF/s)	Cell metabolic activity (e.g., MTT assay)	Color change (OD450)	Color change	Permeation rate (e. g., lidocaine)
**Instrument**	Capacitive sensor (A&A Cap-S)	Spectrometer	Spectrometer	Spectrometer	HPLC
**Complexity**	Simple, uses capacitive sensor	Extremely complex, , requires reagent, assay plate and cell incubations	Complex, requires reagent, assay plate and analysis	Complex, requires reagent, assay plate and analysis	More complex, requires diffusion cells and analysis

**Cost and Time**	Likely lower cost, faster measurement (< 1hr)	Extremely high cost, Extremely time-consuming (>72 hrs)	Higher cost, Time-consuming (24-48 hrs)	High cost, Time-consuming (24-48 hrs)	Higher cost, longer due to analytical chemistry (24-48 hrs)
**Relevance to Irritation**	Indirect, measures barrier integrity	Indirect, measures cell viability	Indirect, measures barrier integrity	Indirect, measures barrier integrity	Indirect, relates to penetration potential
**Biological Relevance**	Limited, synthetic model	Cell model	Limited, synthetic model	Limited, synthetic model	Limited, synthetic model
